# Activity/exercise-induced changes in the liver transcriptome after chronic spinal cord injury

**DOI:** 10.1038/s41597-019-0087-5

**Published:** 2019-06-13

**Authors:** Julia H. Chariker, Sujata Saraswat Ohri, Cynthia Gomes, Fiona Brabazon, Kathryn A. Harman, Kathryn M. DeVeau, David S. K. Magnuson, Michal Hetman, Jeffrey C. Petruska, Scott R. Whittemore, Eric C. Rouchka

**Affiliations:** 1 Department of Neuroscience Training, University of Louisville, 522 East Gray St., Louisville, KY 40202 USA; 2 Kentucky Biomedical Research Infrastructure Network Bioinformatics Core, University of Louisville, 522 East Gray St., Louisville, Kentucky 40202 USA; 3 Kentucky Spinal Cord Injury Research Center, University of Louisville, 511 South Floyd St., Louisville, KY 40202 USA; 4 Department of Neurological Surgery, University of Louisville, 220 Abraham Flexner Way, Suite 1500, Louisville, KY 40202 USA; 5 Department of Anatomical Sciences and Neurobiology, University of Louisville, 511 South Floyd St., Louisville, KY 40202 USA; 6 Department of Health & Sport Sciences, University of Louisville, 2100 South Floyd Street, Louisville, KY 40208 USA; 7 Department of Computer Engineering and Computer Science, Speed School of Engineering, University of Louisville, Duthie Center for Engineering, 2301 South 3rd St., Louisville, Kentucky 40292 USA; 8 Present Address: Wiley Publishing, Hoboken, NJ 07030 USA

**Keywords:** Transcriptomics, Spinal cord injury

## Abstract

Multi-organ dysfunction is a major complication after spinal cord injury (SCI). In addition to local injury within the spinal cord, SCI causes major disruption to the peripheral organ innervation and regulation. The liver contains sympathetic, parasympathetic, and small sensory axons. The bi-directional signaling of sensory dorsal root ganglion (DRG) neurons that provide both efferent and afferent information is of key importance as it allows sensory neurons and peripheral organs to affect each other. SCI-induced liver inflammation precedes and may exacerbate intraspinal inflammation and pathology after SCI, which may be modulated by activity and exercise. In this study, we collected comprehensive gene expression data through RNA sequencing of liver tissue from rats with chronic SCI to determine the effects of activity and exercise on those expression patterns. The sequenced data are of high quality and show a high alignment rate to the Rn6 genome. Gene expression is demonstrated for genes associated with known liver pathologies. UCSC Genome Browser expression tracks are provided with the data to facilitate exploration of the samples.

## Abstract (summary)

**Table:** 

Design Type(s)	gene expression analysis objective • stimulus or stress design • transcription profiling design
Measurement Type(s)	gene expression
Technology Type(s)	RNA sequencing
Factor Type(s)	Spinal Cord Injury • Exercise • EnvironmentalVariable • biological replicate
Sample Characteristic(s)	Rattus norvegicus • liver

Machine-accessible metadata file describing the reported data (ISA-Tab format)

## Background & Summary

More than 10,000 people in the U.S. suffer from traumatic spinal cord injury (SCI) yearly, and the number of people living with paralysis is ~5.4 million^1^. Beyond impairments to sensation and voluntary movement, SCI disturbs the sensory and autonomic nervous systems (ANS) and induces dysfunction in multiple organ systems^2^. Neurogenic pain, depression, cardiovascular disease, liver damage, kidney dysfunction and urinary tract infection are all common in SCI patients and hinder functional recovery and affect long term quality of life. These SCI-induced changes, compounded by a sedentary lifestyle, lead to obesity and a host of metabolic changes, with ~55% of SCI individuals developing frank metabolic syndrome^3^. SCI also triggers a systemic inflammatory response (SIR) that contributes to a high incidence of secondary organ complications^4–7^. Several studies have suggested that post-SCI, the liver contributes to the initiation and propagation of the SIR^4,7,8^. Rodent studies show that traumatic SCI triggers neutrophil infiltration, macrophage activation, and expression of pro-inflammatory cytokines and chemokines in the liver which amplifies the central nervous system (CNS) response to injury^4^. Specifically, liver Kupffer cells control the magnitude of the inflammatory response in the injured brain and spinal cord^8^. This inflammation in the liver appears as early as 30 minutes after injury^9^ and its severity is correlated with lesion level^10^. Further, various liver abnormalities have been observed chronically in humans and rodents post-SCI including non-alcoholic fatty liver disease (humans)^11^ as well as considerable hepatic lipid accumulation (rodents)^12^. These liver pathologies are sustained chronically post-SCI.

Several clinical studies suggest that physical activity can be effective in enhancing recovery of function and also in ameliorating SCI-induced cardiometabolic syndrome, risks of dyslipidemia, and insulin resistance in humans^13–16^. However the underlying mechanisms responsible for these outcomes largely remain unknown. Previous studies have suggested that spontaneous improvements in locomotor function are related to “in-cage activity”^17–20^, indicating that this activity acts as a type of ‘rehabilitative therapy’. This is important because human SCI patients are highly restricted in their activity levels post-SCI. The main objective of this study is to examine the chronic transcriptomic changes in rat liver after SCI and evaluate how this profile is modified with spontaneous in-cage activity and specific forms of exercise, with the premise that these changes may suggest mechanisms.

**Fig. 1:**
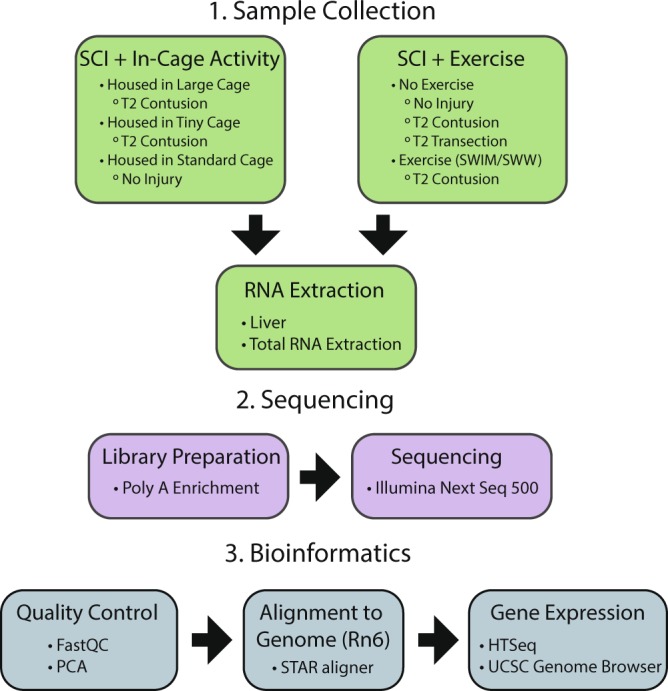
Experimental workflow. (1) In the SCI + In-Cage Activity sample set (top left), rats with CONT injury were housed in large or tiny cages. Rats with no SCI were housed in standard size cages. In the SCI + Exercise sample set (top right), rats received a CONT SCI, a TX SCI, or no SCI. A subset of rats with CONT SCI were exposed to swimming (SWIM) or shallow water walking (SWW) after injury. For both sample sets, RNA was extracted from the liver. (2) RNA samples were subjected to poly A enrichment and sequenced on an Illumina NextSeq 500. (3) The bioinformatics workflow included quality control analysis, alignment to the *Rattus norvegicus* (Rn6) reference assembly, and sample expression analysis with UCSC Genome Browser visualization.

**Fig. 2:**
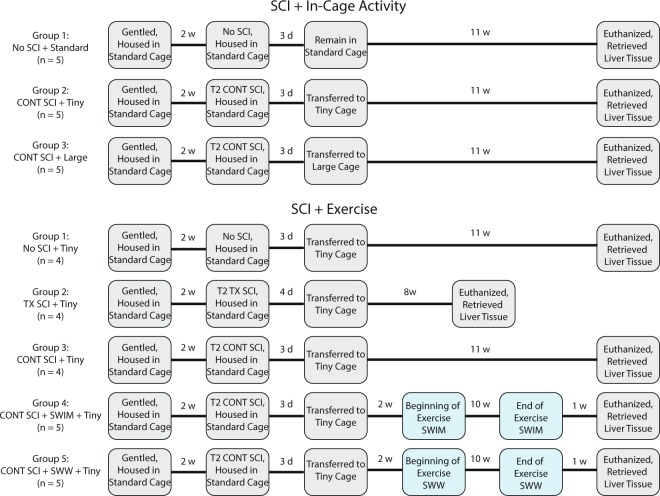
Experimental design. Time course for the SCI + In-Cage Activity groups (top) and the SCI + Exercise groups (bottom).

An overall workflow of the study is schematically represented in Fig. 1 with our experimental design given in greater detail in Fig. 2. For this initial look at the effect of activity/exercise on SCI, we chose female adult rats (~8–9 weeks old) to control for transcriptomic responses related to age and gender. In our experience, female rats recover more quickly from surgery and have greater motivation to exercise, providing our best chance for identifying transcriptomic changes related to activity/exercise. We used two approaches to explore the effect of activity and exercise on injury. In our initial approach, referred to as SCI + In-Cage Activity, we obtained liver samples from contusion (CONT) injured rats housed in large (activity-enhanced) or tiny (activity-restricted) cages. In the latter, in-cage activity is reduced by 75–80% versus large cages for both SCI and naïve groups (DSKM, unpublished observations). The tiny, activity-restricted cages mimic the clinical situation in which sedentary behaviors are enhanced, thereby contributing to the general decline in physical health. In our second approach, referred to as SCI + Exercise, we obtained liver samples from rats housed in activity-restricted (tiny) cages that received one of two different exercise training paradigms: swimming (SWIM) or shallow water walking (SWW), each with its own range of beneficial effects in terms of recovery of function^19–22^. In addition, another group of animals received a complete spinal cord transection (TX) to examine the transcriptome in the total absence of descending, supraspinal innervation.

**Fig. 3:**
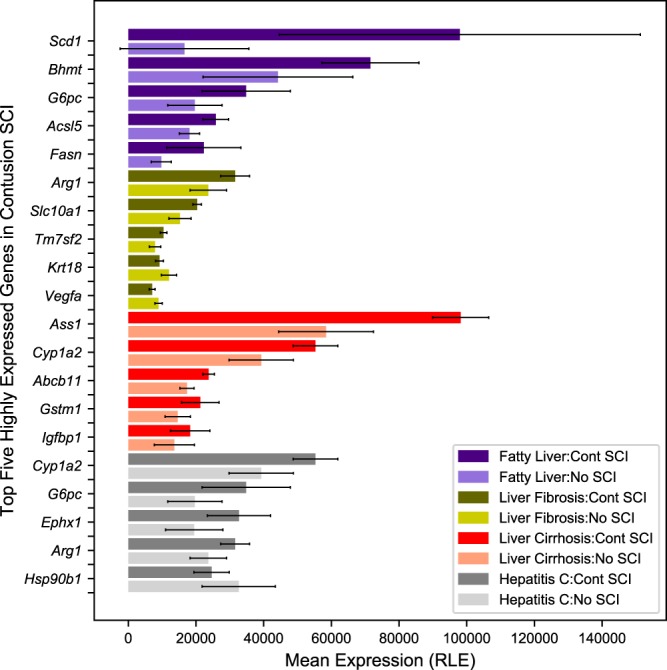
Gene expression in categories relevant to SCI-induced liver pathologies. Five genes associated with four known liver pathologies with the highest mean expression in liver from animals with CONT SCI (with activity restriction by housing in tiny cages) are presented (taken from the SCI + Exercise set). Mean expression for No SCI is included as a comparison. Read counts are normalized using DESeq2’s default method, relative log expression (RLE).

**Fig. 4:**
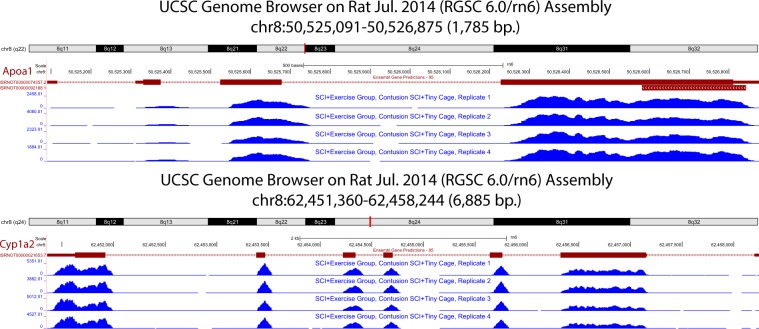
UCSC Genome Browser gene expression tracks. Custom tracks display expression for *Apoa1* (**a**) and *Cyp1a2* (**b**) in four CONT SCI samples (taken from the SCI + Exercise set, tiny cages).

A quality control analysis of our data indicates high quality sequenced reads with high alignment rates to the *Rattus norvegicus* genome (Rn6). Gene expression data indicate high levels of gene activity from categories relevant to SCI-induced liver pathologies (see Fig. 3 for mean expression in CONT injury and No SCI). To facilitate exploration of expression across samples, UCSC Genome Browser^23^ tracks were created and made available with this dataset. In Fig. 4, expression for two enzymes with a specific role in lipid metabolism, apolipoprotein A1 (*Apoa1*) (Fig. 4a) and cytochrome P450 1A2 (*Cyp1a2*) (Fig. 4b) is displayed for CONT SCI samples. The raw data presented here are offered as a valuable resource to the scientific community for future investigation and further elucidation of underlying biological pathways related to SCI-induced pathogenicity in the liver.

## Methods

### Animals

All animal procedures were performed in accordance with the Public Health Service Policy on Humane Care and Use of Laboratory Animals (Institute of Laboratory Animal Resources, National Research Council, 1996) and the University of Louisville Institutional Animal Care and Use Committee. Female Sprague Dawley rats of body weight 235–249 g were obtained from Sprague Dawley, Inc. (Indianapolis, IN). All rats were initially housed in standard cages and maintained in a 12h-light/dark cycle throughout. Tap water and a standard rodent diet were available to all rats *ad libitum*.

### Experimental design and SCI

The experimental workflow from sample collection through sequencing and bioinformatics is displayed in Fig. 1. The experimental design is illustrated in Fig. 2. Prior to the study, 15 animals in the SCI + In-Cage Activity study were randomly divided into three groups: no injury housed in standard cages (No SCI + Standard, 5 replicates), T2 contusion injury housed in tiny cages (CONT SCI + Tiny, 5 replicates), and T2 contusion injury housed in large cages (CONT SCI + Large, 5 replicates). In the SCI + Exercise study, 22 animals were randomly assigned to five groups: no injury (No SCI, 4 replicates), T2 transection injury (TX SCI, 4 replicates), T2 contusion injury (CONT SCI, 4 replicates), T2 contusion injury followed by swimming as exercise (CONT SCI + SWIM, 5 replicates), and T2 contusion injury followed by shallow water walking as exercise (CONT SCI + SWW, 5 replicates). Throughout the study, rats were doubly-housed with individuals from the same experimental group.

All rats were initially gentled for two weeks, during which time they were introduced and acclimated to the testing and exercise facilities. After this period, animals were anaesthetized with a ketamine (50 mg/kg)/xylazine (0.024 mg/kg)/acepromazine (0.005 mg/kg) cocktail (IP) and given glycopyrolate (0.08 mg/kg, IM) prior to SCI surgeries. For all injury groups (CONT and TX), a dorsal midline incision was made in the superficial muscle overlying the T1–T3 vertebrae. A single level laminectomy was then performed at the T2 vertebrae. Animals in the CONT groups received a moderately severe contusion injury (25 g-cm SCI) at the T2 spinal cord level using the NYU Impactor^24,25^. For animals in the TX group, a scalpel was used to deliver a complete transection of the spinal cord at T2. The muscle and skin overlying the injury were sutured in layers and antibiotic ointment was applied to the incision. Injured animals were monitored on heating pads until they recovered from the anesthesia. Rats were then doubly-housed in cages with ALPHA-dri® bedding (Shepherd’s^TM^ Specialty Paper, Milford, NJ) for the remainder of the study. Post-operative care consisted of daily injections of gentamicin sulfate for 7 days (20 mg/kg, S.C.), twice-daily injections of buprenorphine for 3 days (0.03 mg/kg, S.C., and as needed for pain management thereafter), and twice-daily 5 ml boluses of lactated ringers for three days (and as needed for hydration thereafter). Manual bladder expression was conducted three times a day until reflexive voiding was re-established. Rats were maintained on a 12-hour day/night light cycle throughout and had access to standard rat chow and water ad libitum. During the 2 week gentling and a 3 day recovery period, all animals were doubly housed in standard cages, measuring 22″ × 12.5″ × 8″.

Three days after injury, animals in the SCI + In-Cage Activity study were doubly housed in tiny cages (7.5″ × 8.5″ × 8″) to restrict movement and activity or large cages (14″ × 18″ × 8″, base dimension; 16″ × 20″ ceiling) to allow for greater movement for the duration of the study. No SCI controls remained in standard cages.

Three days after injury, all animals in the SCI + Exercise study were doubly-housed in tiny cages to restrict in-cage activity for the duration of the study. Animals in the CONT SCI + SWIM and CONT SCI + SWW groups began exercising 14 days post-injury. Exercise sessions were conducted 5 consecutive days/week for 10 weeks. Animals exercised for 30 minutes each day with 15 minutes of exercise in the morning and 15 minutes in the afternoon. The morning and afternoon sessions were separated by a minimum of one hour. Each 15 minute session consisted of three five minute periods of exercise with breaks between the periods lasting approximately 20–25 minutes.

### Tissue collection and RNA extraction

Animals were sacrificed with a ketamine overdose at 8.5, 11.5, or 13.5 weeks post-SCI, depending on condition (see Fig. 2). All uninjured animals were sacrificed at a time point equivalent to 11.5 weeks post-SCI in the other animals. Hearts were arrested in diastole with an injection of 3 M KCl. Animals were perfused with PBS supplemented with 20% RNA *later* (Ambion, Life Technologies, Carlsbad, CA). Livers were taken from each animal and 200 mg of liver tissue was processed from each using RNeasy Lipid Tissue Mini Kit (Qiagen) to isolate RNA.

### Library preparation and sequencing

1 µg of total RNA samples were used for poly A enrichment. First and second strands were synthesised followed by 3′ end adenylation. Samples were barcoded with Illumina TrueSeq adapters. 1.8 pM of barcoded library was denatured, and sequencing was performed on the University of Louisville Center for Genetics and Molecular Medicine (CGeMM) Illumina NextSeq 500 using the NextSeq 500/550 1X75 cycle High output kit (Illumina, Carlsbad, CA).

### RNA-seq data analysis

Sequencing produced over 1 billion single end reads across the 37 samples. The vast majority of read lengths fell between 74–76 bases across all samples. The quality of the reads was assessed using FastQC v.0.10.1^26^, which indicated no sequence trimming was necessary. The sequences were directly aligned to the *Rattus norvegicus* reference genome assembly (Rn6) using Star version 2.6^27^. Read counts for gene regions were obtained with HTSeq (version 0.10.0)^28^ using Ensembl annotations^29^ (Rn6 version 93). The annotation file was parsed to exclude mitochondria genes in an effort to reduce non-relevant variation in subsequent steps of the analysis. The resulting annotation file extracted read counts for 24,613 gene locations.

A principal component analysis (PCA) was performed using the R programming language^30^ to examine within- and between-group variability of the samples. Three-dimensional PCA plots were generated using the R package ‘pca3d’. DESeq2’s regularized log transformation^31,32^ was applied to the raw counts prior to PCA to reduce the effect of high level variation genes on the spread of sample points.

Prior to examining gene expression, raw read counts were normalized to remove natural variation across samples arising from differences in tissue sampling and sequencing using DESeq2’s default method, relative log expression (RLE)^31–33^. UCSC Genome Browser tracks were created to facilitate exploration of gene expression in each of the samples^23^. The tracks were created using methods and available utilities described on the UCSC Genome Browser website for converting sequencing alignment files in BAM format to BigWig format.

## Data Records

The data were submitted to NCBI Gene Expression Omnibus (GEO; GSE124819)^34^. This GEO project includes raw data in Fastq format, raw HTSeq counts^28^, and UCSC Genome Browser tracks in bigwig format for all samples^23^. This dataset is part of a larger study measuring the systemic transcriptional response to spinal cord injury, including dorsal root ganglion^35^ and soleus muscle, all of which are included as part of a GEO superseries (GSE129704)^36^.

## Technical Validation

### RNA metrics

Sequencing generated 26.5 to 46.8 million reads/sample with a mean of 37.1 million and standard deviation of 6.3 million. Table 1 displays the number of raw reads successfully aligned for each of the samples. The alignment rate for uniquely mapped and multi-mapped reads combined ranged from 85.97 to 98.87 percent with a mean of 97.85 across the 37 samples.

**Table 1:** Sequencing and alignment summary.

Sample ID	Input Reads	Number Uniquely Mapped Reads	Percent Uniquely Mapped Reads	Number Multi-mapped Reads	Percent Multi-mapped Reads
SCI + Exercise Group, No SCI + Tiny Cage, Replicate 1	42,399,033	37,397,063	88.20%	4,454,134	10.51%
SCI + Exercise Group, No SCI + Tiny Cage, Replicate 2	43,891,878	39,065,260	89.00%	4,273,932	9.74%
SCI + Exercise Group, No SCI + Tiny Cage, Replicate 3	39,487,917	35,029,130	88.71%	3,995,282	10.12%
SCI + Exercise Group, No SCI + Tiny Cage, Replicate 4	46,599,189	41,202,534	88.42%	4,807,627	10.32%
SCI + Exercise Group, Contusion SCI + Tiny Cage, Replicate 1	44,632,283	39,473,950	88.44%	4,654,653	10.43%
SCI + Exercise Group, Contusion SCI + Tiny Cage, Replicate 2	44,373,773	39,529,977	89.08%	4,321,664	9.74%
SCI + Exercise Group, Contusion SCI + Tiny Cage, Replicate 3	36,792,367	32,526,721	88.41%	3,831,897	10.41%
SCI + Exercise Group, Contusion SCI + Tiny Cage, Replicate 4	40,831,557	36,185,025	88.62%	4,164,017	10.20%
SCI + Exercise Group, Contusion SCI + SWW + Tiny Cage, Replicate 1	45,075,405	40,158,028	89.09%	4,219,134	9.36%
SCI + Exercise Group, Contusion SCI + SWW + Tiny Cage, Replicate 2	41,664,670	36,976,616	88.75%	3,777,884	9.07%
SCI + Exercise Group, Contusion SCI + SWW + Tiny Cage, Replicate 3	39,537,199	35,356,379	89.43%	3,500,321	8.85%
SCI + Exercise Group, Contusion SCI + SWW + Tiny Cage, Replicate 4	43,921,348	39,440,686	89.80%	3,823,873	8.71%
SCI + Exercise Group, Contusion SCI + SWW + Tiny Cage, Replicate 5	45,886,655	40,428,408	88.10%	4,584,917	9.99%
SCI + Exercise Group, Complete Transection + Tiny Cage, Replicate 1	41,554,722	36,502,828	87.84%	4,382,702	10.55%
SCI + Exercise Group, Complete Transection + Tiny Cage, Replicate 2	40,509,133	34,679,508	85.61%	5,070,415	12.52%
SCI + Exercise Group, Complete Transection + Tiny Cage, Replicate 3	39,287,418	34,572,792	88.00%	3,962,347	10.09%
SCI + Exercise Group, Complete Transection + Tiny Cage, Replicate 4	40,271,252	33,875,382	84.12%	4,622,422	11.48%
SCI + Exercise Group, Contusion SCI + Swim + Tiny Cage, Replicate 1	36,686,399	31,595,516	86.12%	3,835,657	10.46%
SCI + Exercise Group, Contusion SCI + Swim + Tiny Cage, Replicate 2	41,969,824	37,158,530	88.54%	3,678,357	8.76%
SCI + Exercise Group, Contusion SCI + Swim + Tiny Cage, Replicate 3	39,865,248	30,349,730	76.13%	3,922,770	9.84%
SCI + Exercise Group, Contusion SCI + Swim + Tiny Cage, Replicate 4	46,805,595	41,263,700	88.16%	4,525,924	9.67%
SCI + Exercise Group, Contusion SCI + Swim + Tiny Cage, Replicate 5	39,661,789	33,517,408	84.51%	4,013,227	10.12%
SCI + In-Cage Acitivity Group, Contusion SCI + Large Cage, Replicate 1	29,044,542	25,743,435	88.63%	2,877,489	9.91%
SCI + In-Cage Acitivity Group, Contusion SCI + Large Cage, Replicate 2	30,149,297	26,311,763	87.27%	3,176,905	10.54%
SCI + In-Cage Acitivity Group, Contusion SCI + Large Cage, Replicate 3	29,172,739	25,664,008	87.97%	3,018,588	10.35%
SCI + In-Cage Acitivity Group, Contusion SCI + Large Cage, Replicate 4	31,531,038	27,647,614	87.68%	3,398,095	10.78%
SCI + In-Cage Acitivity Group, Contusion SCI + Large Cage, Replicate 5	30,336,893	26,937,035	88.79%	2,955,981	9.74%
SCI + In-Cage Activity Group, Contusion SCI + Tiny Cage, Replicate 1	31,803,908	28,148,074	88.51%	3,204,594	10.08%
SCI + In-Cage Activity Group, Contusion SCI + Tiny Cage, Replicate 2	32,829,085	28,841,325	87.85%	3,384,185	10.31%
SCI + In-Cage Activity Group, Contusion SCI + Tiny Cage, Replicate 3	30,176,837	26,630,179	88.25%	3,121,377	10.34%
SCI + In-Cage Activity Group, Contusion SCI + Tiny Cage, Replicate 4	33,007,402	29,196,209	88.45%	3,322,693	10.07%
SCI + In-Cage Activity Group, Contusion SCI + Tiny Cage, Replicate 5	26,517,090	23,395,916	88.23%	2,728,068	10.29%
SCI + In-Cage Activity Group, No SCI + Standard Cage, Replicate 1	27,726,412	24,509,335	88.40%	2,860,220	10.32%
SCI + In-Cage Activity Group, No SCI + Standard Cage, Replicate 2	32,117,272	28,086,368	87.45%	3,538,659	11.02%
SCI + In-Cage Activity Group, No SCI + Standard Cage, Replicate 3	31,552,450	27,869,954	88.33%	3,143,354	9.96%
SCI + In-Cage Activity Group, No SCI + Standard Cage, Replicate 4	27,922,944	24,366,835	87.26%	3,098,243	11.10%
SCI + In-Cage Activity Group, No SCI + Standard Cage, Replicate 5	29,351,626	26,074,290	88.83%	2,885,046	9.83%

### Quality assessment

**Fig. 5:**
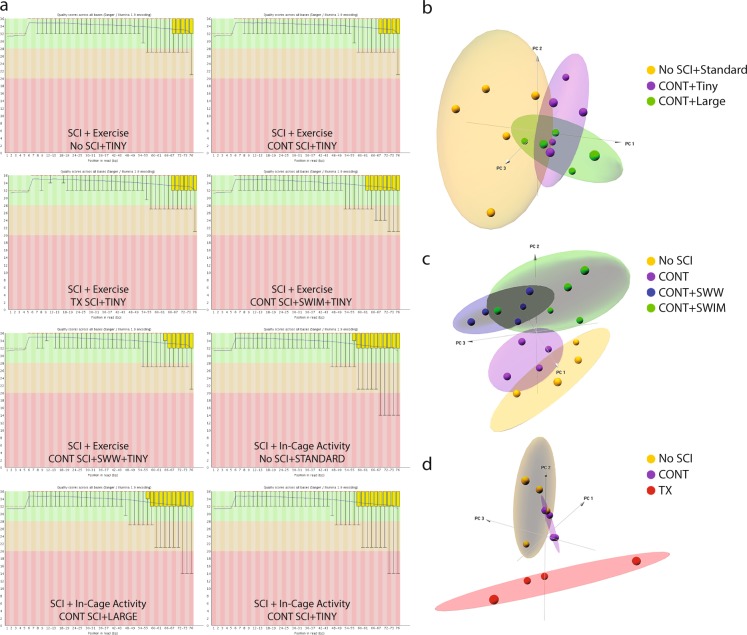
Quality control analysis. Phred quality scores per base for one representative sample from each experimental group. (**a**) On the Y-axis a Phred score of 30 indicates 99.9% accuracy in base calling. Phred scores above 28 (green) are considered very good quality. Scores between 20 and 28 (orange) are considered reasonable quality. Scores below 20 (red) are considered poor quality. The yellow box represents the inter-quartile range (25–75%). The lower and upper whiskers represent the 10^th^ and 90^th^ percentiles respectively. On the right, 3D PCA plots for SCI + In-Cage Activity samples (**b**) SCI + Exercise samples minus TX SCI (**c**) and PCA focused solely on a comparison of injury severity (**d**).

Raw sequencing data was input to FastQC for quality assessment. All samples were deemed of high quality. In Fig. 5a, the Phred quality score per base is displayed for a representative sample from each experimental group. For all samples, the 25^th^ percentile of quality scores is at or above a Phred score of 30, reflecting 99.9 percent accuracy in base calling. The gradual drop in quality at the end of the sequence is a common phenomenon with Illumina’s approach to sequencing by synthesis^37^.

### Gene expression variation of biological replicates

PCA was performed to assess the within- and between-group variation of the samples. In Fig. 5b–d, three-dimensional PCA plots provide a view of sample points in 3D space. In Fig. 5b, the SCI + In-Cage Activity samples appear to have large variability within experimental groups (Variance: PC1 29%, PC2 13%, PC3 11%), reflecting some degree of individual differences across animals, one possible source being variability in the spontaneous activity of animals regardless of cage size. Importantly, however, the No SCI samples appear in a distinct region of the plot from the CONT SCI samples. A subset of samples from the CONT + Tiny and the CONT + Large groups are clearly distinct from each other with a few samples overlapping. The number of samples in each group, five each, would allow for removal of overlapping samples with a sufficient number of samples remaining, at least three, for comparison.

In Fig. 5c, PCA was performed to examine the separation between CONT SCI alone and CONT SCI followed by exercise (Variance: PC1 32%, PC2 13%, PC3 10%). The CONT + SWIM and CONT + SWW groups appear to overlap somewhat but are located in a distinct region of the graph from the CONT SCI and No SCI groups. Once again, sufficient samples are available in the SCI + Exercise groups to allow the removal of any overlapping samples between the SWW and SWIM groups. The CONT SCI samples cluster well and appear separate from the No SCI samples.

In Fig. 5d, PCA was performed to look at the separation between CONT SCI and TX SCI samples (Variance: PC1 28%, PC2 26%, PC3 9%). TX SCI samples vary widely within the group, but lie in a distinct location in the graph from the CONT SCI and the No SCI samples. In this case, there appears to be some overlap between the CONT SCI and the No SCI samples. However, by removing one sample from each group in the overlapping region, three samples remain distinctly separate in each group for comparison purposes.

Gene expression was examined to confirm that high level activity was found for genes relevant to liver function. In Fig. 3, the mean expression across CONT SCI samples is displayed for highly expressed genes associated with four well-documented liver pathologies. Mean expression for No SCI is included as a comparison. The genes associated with each category were obtained from topGO^38^. Figure 4 displays the UCSC Genome Browser expression tracks for the CONT SCI samples positioned at two genes known to be involved in lipid metabolism, *Apoa1* (Fig. 4a) and *Cyp1a2* (Fig. 4b). Expression appears consistent at all locations across the four CONT SCI animals.

### Potential batch effects

The length of time between injury and tissue collection varied from 8.5 to 13.5 weeks in an attempt to balance the requirements of our research design with the well-being of the animals. The exercised animals required an initial period of time for introduction into the exercise facility followed by a full 10 weeks of exercise prior to measurement, resulting in tissue collection at 13.5 weeks post-injury. In contrast, the animals with a complete transection of the spinal cord required extensive care to ensure their well-being. Studies have shown that physiological measures plummet and stabilize by four weeks post-injury in the case of transection injury^39^. All batch effects were controlled since tissue was collected by the same individual using the same method and occurred well past the sub-acute to chronic stage transition which generally occurs 4 weeks post-injury^40^.

## ISA-Tab metadata file

Download metadata file

## Data Availability

All analyses were performed using open sources software tools. Raw sequencing files were downloaded from Illumina BaseSpace using the Illumina Python Run Downloader^41^. Individual samples were initially divided across four lanes for sequencing, and these files were concatenated into one single end Fastq file using the UNIX cat command. cat <*FN1*>.fastq <*FN2*>.fastq <*FN3*>.fastq <*FN4*>.fastq> <*COND_REP*>.fastq The concatenated sequences were input to FastQC v.0.10.1^26^ for quality control analysis using default parameters. fastqc <*COND_REP*>.fastq –o <FASTQC_DIRECTORY> Fastq files were input to Star 2.6^27^ for alignment specifying BAM file format sorted by coordinate and requesting unmapped read files. STAR–runMode alignReads–outSAMtype BAM SortedByCoordinate –outSAMstrandField intronMotif–outReadsUnmapped Fastx–readFilesIn <*COND_REP*>.fastq.gz –outFileNamePrefix <*COND_REP*>–runThreadN 16–genomeDir Rnor_6.0 –readFilesCommand zcat Read counts for each sample were extracted using HTSeq 0.10.0^28^. The *reverse* option was used to indicate strand orientation. Illumina’s TruSeq Stranded mRNA protocol was used to sequence the data and produces libraries where the first read is on the opposite strand to the RNA molecule. htseq-count -f bam–stranded=reverse–mode=intersection-nonempty -r name <*COND_REP*>/Aligned.sortedByCoord.out.bam\Rattus_norvegicus.Rnor_6.0.93_PARSED.gtf> <*COND_REP*>/gene_counts_Reversed.htseq The raw counts were normalized using DESeq2’s^31,32^ default procedure, relative log expression (RLE), using the *estimateSizeFactors* function. Detailed instructions can be found on the Bioconductor website for DESeq2^42^.
